# An expenditure analysis revealing how Philip Morris advertisements coincide with tobacco policymaking in Switzerland

**DOI:** 10.18332/tpc/189922

**Published:** 2024-06-28

**Authors:** Kris Schürch, Annika Frahsa, Harvy Joy Liwanag, Luciano Ruggia

**Affiliations:** 1 Institute of Social and Preventive Medicine, University of Bern, Bern, Switzerland; 2 Graduate School for Health Sciences, University of Bern, Bern, Switzerland; 3 Swiss Association for Tobacco Control, Bern, Switzerland

**Keywords:** health policy, WHO FCTC, tobacco, tobacco advertisements

## Abstract

**INTRODUCTION:**

Prior research has linked media tobacco promotion to increased tobacco use and favorable perceptions of tobacco products. Switzerland's tobacco lobby employs advertisements to sway policy decisions in its favor, yet no recent research has assessed this in detail. Our study aims to provide detailed estimates of tobacco industry (TI) advertisement costs, focusing on Philip Morris International (PMI) in Switzerland, and examine potential chronological links between TI advertisement campaigns and parliamentary discussions on tobacco bills. By spreading knowledge on this issue, we aim to support the development of future tobacco advertisement regulations.

**METHODS:**

We conducted an expenditure analysis of tobacco-related press advertisements in Swiss print media published between August 2020 and August 2021, accessed through the media intelligence firm Argus Data Insights. Advertisement costs were estimated using publicly available data. We plotted expenditure sums of PMI against key parliamentary session dates featuring discussions on proposed tobacco control measures, such as tighter restrictions on advertising.

**RESULTS:**

Over 12 months, 501 advertisements with tobacco-specific headlines were published in Swiss press media. Of these, 437 advertisements (87.22%) were linked to PMI. PMI accounted for 88.21% (CHF 6486969) of total advertisement expenditure. Notably, PMI advertisements coincided with key political sessions discussing tobacco legislation in parliament, with a limited presence outside these periods.

**CONCLUSIONS:**

PMI advertisements were published parallel to key moments of parliamentary discussions, suggesting an attempt by TI to potentially influence discussions. Applying such an advertisement monitoring methodology helps understand the contextual conditions of public health in Switzerland. By analyzing TI advertisements in print media, we sought to highlight regulatory gaps and support the creation of stricter advertising regulations. We recommend continuing such research to strengthen tobacco control policymaking. Key public health efforts should include raising awareness of TI tactics, implementing a comprehensive ban on tobacco advertising, and strategically engaging with the media in tobacco control campaigns.

## INTRODUCTION

Switzerland, home to three major multinational tobacco corporations – Philip Morris International (PMI), British American Tobacco, and Japan Tobacco International, remains under-regulated with regard to tobacco advertising^1^. From a public health perspective, this insufficient regulation is critical, as advertising serves as a potent tool that tobacco firms employ to influence consumers, particularly at a young age, concerning their perception of tobacco products and tobacco use^2-5^.

The tobacco industry (TI) is known for its strategies to circumvent public health policies to ensure they may keep persuading the public to continue smoking. For example, tobacco companies have developed various strategies to manufacture doubt and legitimize their role as stakeholders in science and policymaking to divert public and policymaker attention away from health issues their products cause^6-10^. Recent studies by Goldberg and Vandenberg^11^ and Legg et al.^12^ summarized TI strategies to veil their efforts, such as working with consultancy firms, politicians with industry ties, as well as media outlets to defend standard industry arguments. In the past, such TI strategies have given rise to international action, with the World Health Organization Framework Convention on Tobacco Control (WHO FCTC) – currently ratified by 182 countries – translating evidence of TI activity into preventive policy action^13-15^. Very few countries – mostly tobacco-growing states – have not ratified WHO FCTC. Switzerland has not ratified the WHO FCTC since signing it in 2004^16^.

### Switzerland as a case study

Switzerland presents a unique and compelling research opportunity due to its liberal market economy, the concentration of TI power, and its robust liberal democracy, which often places individual and entrepreneurial freedoms at the forefront at the expense of public health^17,18^.

Swiss media legislation protects fundamental freedoms and places restrictions on governmental power to stifle media independence (Swiss Federal Constitution Art. 16). The media landscape in Switzerland, influenced by the country’s multilingual setup and direct democracy, is divided into three distinct language regions (French, German, and Italian), each with its own media market and journalistic culture^19^. Media play a crucial role in shaping public opinion and political discussions, especially during frequent public votes where citizens directly influence governmental decisions^20^. Esser et al.^19^ note that the segmentation of these small media markets, with limited financial resources, makes them susceptible to external influences, notably affecting public perception and policy debates^19^. Historical instances in 1979 and 1993 demonstrate how an effective coalition formed between the tobacco industry, advertising firms, and print media effectively swayed public opinion against bans on tobacco and alcohol advertising by framing such restrictions as economically detrimental and a threat to job security and free speech^21,22^.

TI influence on Swiss media and policy-making

In 2019, Switzerland ranked second last out of 36 European countries in the Tobacco Control Scale that measures the implementation of tobacco control policies at the country level. Regarding the ‘policy of comprehensive bans on advertising and promotion’, Switzerland even scored last^1^. Similarly, in 2023, Switzerland ranked second last in the Global Tobacco Industry Interference Index – an international tobacco lobby index aligned with the guidelines under Article 5.3 of the FCTC – measuring government efforts to tackle TI influence. Switzerland, thus, is considered a particularly industry-friendly nation where tobacco companies experience little resistance to their campaigns^23,24^.

On 1 October 2021, a tobacco product law – likely entering into force at the end of 2024 – was approved following a string of parliamentary discussions. The law introduces only marginal changes with insignificant effects for improving public health and contributes to ranking Swiss tobacco control legislation as the weakest in Europe. Tobacco advertisements in print or online media – largely consumed by younger age groups – remain allowed^25^. The persistence of such advertisements, especially those targeting younger people, underscores a global pattern where TI tactics influence political and legislative outcomes. Often, tobacco advertisement campaigns also coincide with political debates, such as in Argentina, South Africa, or the United States^26,27^.

In Switzerland, Lee and Glantz^21^ found a similar link; they showed TI in Switzerland led well-organized, often disguised media campaigns with advertising agencies and print media to sway Swiss voters to reject tobacco and alcohol advertising bans in 1979 and 1993. Lee and Glantz^21^ stated TI possesses ‘almost unlimited financial resources’, yet they did not provide estimates of TI advertisement costs in Switzerland. Such estimations of expenditure costs can be useful as they quantify the financial resources dedicated to influencing public opinion. These data can reveal the intensity of tobacco advertising campaigns and pinpoint where regulations may be needed, particularly when adding a focus on policymaking sessions rather than public votes and integrating new tobacco products that have been introduced since the paper by Lee and Glantz^21^.

### Aims of the study

In our study, we thus aim to provide detailed estimates of TI advertisement costs in Switzerland, as well as to examine patterns between TI advertisement campaigns and parliamentary discussions surrounding the recent tobacco product law. Our focused analysis on PMI advertisements is driven by an observed uptake in PMI-related advertising activities during the study period relative to other tobacco entities. This pattern, coupled with PMI’s well-documented history of engaging in efforts to influence public policy and legislative outcomes, prompted us to delve deeper into their advertising tactics to understand their potential impact on policy discussions in Switzerland. By profiling and analyzing TI advertisements in print media, we aim to shed light on an existing regulatory gap in Switzerland and offer policymakers an evidence-based foundation to inform and advocate for future tobacco advertisement regulations.

## METHODS

### Data collection

We retrieved tobacco-related print advertisements (TA) printed in Swiss press media between a twelve-month period from 18 August 2020 and 30 August 2021 from Argus Data Insights services, one of the leaders providing media coverage with comprehensive print media data sets in Switzerland^28^. Online advertisements are not collected by Argus Data Insights.

We collected cost estimates of all advertisements using official rates provided by media outlets either via their websites or websites of media advertisement company Goldbach Group AG; they market and place advertisements in Switzerland^29^. Goldbach Group acts as a consultancy, handles media campaigns, and works closely with various major media outlets such as *20 Minuten, 24 Heures, and Berner Zeitung,* to name a few. Published rates on different media outlet websites clearly stated prices for various sizes and color ratios (black and white advertisements cost less than advertisements in color), with specifications for printed press or online advertisements. We used published rates to determine expenditures companies incurred across various media outlets.

### Data analysis

We exported the data set into Microsoft Excel for processing as individual Spreadsheet files. The Argus Data Insights database had ascribed each advertisement to its media outlet publisher, along with publication date, headline, language, edition number, type of media, color base, and advertisement size (millimeters). The headlines, which described the topic or subject of the advertisement, were pre-ascribed by the Argus Data Insights database. Moreover, the Argus Data Insights database included a digital scan link. We filtered the data set to only include advertisements that were exclusively advertising a tobacco product with tobacco industry-related headlines, which were identifiable by tobacco-related headlines, such as IQOS, Davidoff Cigarettes, Philip Morris, or Epok. Therefore, we excluded advertisements from large retailers that paid to include tobacco products alongside a broader range of items, often including produce or food, in their advertisements.

For cost analyses of the print advertisements, we matched advertisement image area (height × width) with relevant rates from particular media outlets. All costs were provided and calculated in Swiss Francs (CHF). We converted CHF into United States Dollars (average exchange rate in 2020 was 1 CHF to 1.0665 USD).

In a subsequent step, we separated advertisements related to PMI from other TAs since it became clear most TAs were PMI (Table 1). Thus, to further explore the presence of PMI-relevant advertisements, we only included ‘IQOS’ and ‘Philip Morris’ headlines in our final data set. It helped us specifically track PMI expenses and compare them with others in the data set. We summed all calculated expenditures across printed press by calendar week, print outlet, and headline. Finally, we graphically illustrated sum costs with indications of parliamentary sessions on tobacco product laws during data set periods.

**Table 1:** Distribution, number, and calculated expenditures of tobacco-related advertisements (TA) in Swiss Francs (CHF) and United States Dollars (USD)

*Headlines (brands)*	*Number of advertisements*	*Expenditure (CHF)*	*Percent of TA expenditure*	*Expenditure (USD)*
IQOS (product ad)	328	3992366	61.54	4257858.34
PHILIP MORRIS (non-product, political ad)	109	1729851	26.67	1844886.09
Davidoff	39	492297	7.59	525034.75
ZYN	6	99870	1.54	106511.36
Swedish Match	3	49935	0.77	53255.68
Coop	3	26100	0.40	27835.65
Epox	3	26100	0.40	27835.65
Davidoff Cigars	1	18000	0.28	19197
ASHTON	1	6900	0.11	7358.85
ANIVERSARIO SAMANA	1	6900	0.11	7358.85
PARORO	1	6900	0.11	7358.85
Psyko SEVEN	1	6900	0.11	7358.85
WELLAUER	1	6900	0.11	7358.85
INTERTABAK AG	1	6900	0.11	7358.85
NORDICSPIRIT.CH	1	6050	0.09	7358.85
FRATELLO CIGARS	1	2500	0.04	2666.25
zigarrenversand.ch	1	2500	0.04	2666.25
**Exclusive TA total**	501	6486969		6918352.44
**TA in data set from Argus Data Insights**	792	8582226		9152944.03

One author (KS) screened PMI advertisements and subsequently categorized them into two groups, namely, by advertising PMI’s flagship heated tobacco product (HTP) IQOS or not.

## RESULTS

We present results for the following: 1) TA distribution, 2) TA expenditures, and 3) PMI advertisement type and expenditure results.

### TA distribution

In the printed press data set from Argus Data Insights, there were 792 items; 501 (63.26 %) were TA, which exclusively advertised a tobacco product with a tobacco-related headline. Among the 501 exclusive TA, we traced 437 (87.22 %) back to PMI (headlines IQOS and Philip Morris). Advertisements were all displayed in color, ranged from one-quarter to one page, and were placed in 28 different print media outlets in Switzerland. The official rates of advertisements for 15 print media outlets were accessed through Goldbach Group AG, while the rest were accessed through the websites of individual media outlets (Table 1).

### TA expenditures

Exclusive TA amounted to 75.59% (CHF 6486969) of the total estimated costs for all printed press advertisements in the data set (CHF 8582226). Among exclusive TA costs, 88.21% (CHF 5722217) were PMI-related advertisements. Similarly, the majority of PMI advertisements ranged from one-quarter-page to one-page advertisements, yet also included one two-page advertisement (Table 1).

### PMI advertisement types and expenditures

Among PMI-related TA, we identified one fundamental difference between the PMI headlines – IQOS and Philip Morris. The former advertised only PMI’s IQOS product (https://tobaccotactics.org/wiki/iqos-use-evidence/), while the latter did not advertise a product at all. We categorized these into two types of advertisements: political advertisements without products (109) and product advertisements (328).

Promoted as a healthier alternative to smoking tobacco, PMI product advertisements totaled 70% (CHF 3992366) of total PMI expenditures (CHF 5722217), with political advertisements equaling 30% (CHF 1729851) of the total spent by PMI (Table 1). PMI advertisements were duplicates of themselves, with political advertisements focusing on PMI’s commitment to a smoke-free future and PMI’s use of ‘science’ to improve public health, while product advertisements showed PMI’s IQOS, with minor language variations depending on the language region of the print media outlet.

**Figure 1:**
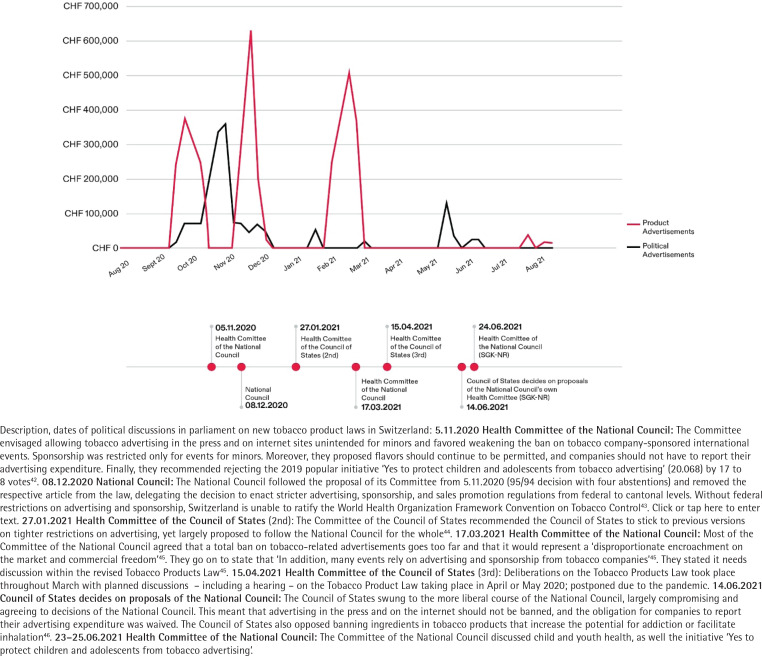
PMI advertisement costs by month with key political sessions on new tobacco product legislation

Our calculated expenditures for PMI-related TA across the study period illustrate trends over time (Figure 1). From September 2020 onwards, we identified four significant expenditure waves with peaks on 25 October 2020 (reaching CHF 376611), 29 November 2020 (reaching CHF 358579), 20 December 2020 (reaching CHF 637706), and 14 March 2021 (reaching CHF 513936). In Figure 1, the separately illustrated PMI product and political advertisements highlight separate PMI marketing strategies that coincide with key parliamentary meetings.

## DISCUSSION

Our analysis revealed that 75.59% (CHF 6486969) of all tobacco printed press advertisement expenditures in Switzerland were exclusive TA. Among exclusive TA costs, 88.21% (CHF 5722217) were PMI-related advertisements. Since the Lee and Glantz^21^ report, our study, to the best of our knowledge, is the first to profile and analyze TA expenditures in printed press in Switzerland, as well as shed light on both product and political advertisements. This study provided insights into PMI’s media strategy to go beyond traditional direct product marketing in attempts to reshape and re-legitimize themselves in the public eye.

Our data revealed that PMI’s advertising campaigns were strategically timed to coincide with political meetings, suggesting attempts to influence Swiss tobacco policymaking discussions. This observation aligns with findings from other research indicating that strategic media campaigns can significantly impact and shape public sentiment towards public health issues^30^.

Our findings suggest a dual purpose in PMI’s advertisements. The politically oriented ones aim to position PMI as a driver of scientific innovation to help build credibility for themselves with the public and – more importantly – policymakers. The absence of a product seems to emphasize an attempt to sell an idea or legitimize PMI as a public health actor. PMI’s commitment to a smoke-free future and their claim as innovators of science align with various TI strategies to help establish credibility, create opportunities with policymakers, and make the industry appear as an important part of development or regulatory efforts^11,12,18^. In contrast, product advertisements aggressively market IQOS to the public, emphasizing its ‘without combustion and smoke’ attributes to convince the public of the ‘healthier nature’ of IQOS^31^.

Although it appears that PMI’s strategic placement of these advertisements around political meetings of various parliamentary bodies seems to point to attempts towards influencing the public and political process, our method does not provide direct evidence of surges in print TA as purposefully synchronized with parliamentary meetings. For example, it is difficult to clearly link how media covered – or kept silent about – political debates due to financial interests linked to TI expenditures.

Nevertheless, considering nearly CHF 6.5 million was spent for exclusive print TA within a one-year period, it reaffirms that the TI is a well-financed, powerful industry that can leverage substantial resources to influence public opinion and policy by creating doubt about the potential harms of their products, as well as legitimize their role as stakeholders in science and society^11,12^. The transition to digital platforms will likely provide the TI with additional opportunities for spreading misinformation and advertising seemingly healthier products^11,32^. Since it became apparent to us that print articles were also available online, it seems PMI have already begun to take advantage of these new online media platforms as well.

Regarding e-cigarette marketing communication channels, a systematic review of e-cigarette marketing communication channels by Lyu et al.^33^ found that among the four communication channels – print, TV/movie/streaming/radio, Internet, and point-of-service/retail stores – the Internet was the most studied channel (78% of included papers)^33^. Thus, while this corresponds with the growing presence of e-cigarette marketing on social media platforms, it indicates a large gap between the amount of marketing expenditure on print and TV, as well as research gaps between products^34-37^.

Although other media platform expenditures remain unclear, the almost unlimited financial resources of TI, as well as the absence of any regulations limiting online advertisements, are a cause of concern since TI has a wide-open playing field to advertise its products and their political ideals in Switzerland. Additionally, without adequate policies limiting print tobacco advertisements, print media essentially develop a dependence on financial earnings tobacco companies provide^19^.

Acknowledging and addressing the gaps in accountability is also essential to address the challenges in tobacco control in Switzerland. Current accountability mechanisms for the TI are notably lacking, highlighting an urgent need to explore and establish effective mechanisms that will hold the tobacco industry, including powerful entities like PMI, to account. These accountability mechanisms should encompass a comprehensive approach, integrating institutional, interinstitutional, and systems-level activities, along with political engagement, to effectively address and mitigate TI influence^38^. Given that Switzerland has failed to ratify the FCTC, we recommend strengthening accountability through mechanisms outside the framework provided by an international convention. For example, civil society organizations play an important role in mobilizing political action to call out attempts by the TI to influence government. In this context, we are also reflexive about our role as scholars of public health and argue that by writing about this topic in the literature, we also seek to heighten awareness about this issue and call into account the unethical practices of the TI in trying to influence policymaking in Switzerland.

### Policy relevance

In terms of policy implications, our findings underscore the inadequacy of current tobacco control measures in Switzerland. The strategic investment by PMI in print advertising, as demonstrated by their extensive expenditure and targeted campaigns, illustrates the ongoing challenge in countering tobacco industry tactics under existing regulations. Article 13 of the WHO FCTC emphasizes the need for comprehensive advertising bans, including non-traditional forms of advertising. Our study suggests that Switzerland needs to strengthen its tobacco control framework to effectively limit industry influence. The financial dependence of media publishers on tobacco advertising obstructs a comprehensive ban on advertising and promotion aligned with Article 13 of FCTC, as is the case in the UK and Australia^39,40^. No significant new tobacco control regulations considered in new tobacco product laws demonstrate the continued successes of TI and PMI deploying their media and lobby tactics. It also highlights the importance of FCTC signees to comply with guidelines to stifle TI’s influence on public health policymaking. In the Swiss context, it is clear that civil society, public health actors, non-governmental organizations, and relevant politicians must work together to ensure Switzerland ratifies WHO FCTC and, in turn, safeguard public health from TI. Crucial public health efforts include raising awareness of TI tactics and ensuring a comprehensive ban on tobacco advertising in Switzerland. Furthermore, the media’s role in shaping public and policy agendas emerges as a critical area for understanding their influence, suggesting that the media not only reflect but also can shape societal norms and policy discussions around tobacco. This parallel highlights the necessity for a nuanced approach to evaluating media influence, acknowledging its potential to both inform and sway public opinion and health-related behaviors, thereby underscoring the importance of strategic media engagement in public health campaigns targeting tobacco use^41^.

### Limitations

A key constraint of our argument is expenditure calculations based on a limited one-year period of data collection. The data set we received from Argus Data Insights only included print advertisements. We did not consider other streams or media, particularly online advertisements, which means our data likely represent the ‘tip of the iceberg’ regarding advertisement expenditures. Since online advertisements are elusive and harder to track, it is more challenging to estimate their costs. Moreover, online advertisements are not limited by national borders and are sometimes funded internationally; it further supports that TI expenditures are likely much greater than we indicated since we merely analyzed expenditures from a single form of advertising. The internet provides a unique platform for multinational tobacco companies to reach huge audiences without investing much time or resources to spread their message^11^. Future research should focus on developing methods to specifically assess online advertisement spending on different tobacco and nicotine products.

Finally, we made our expenditure calculations based on publicly available information. We are unaware of PMI’s exact expenditures during print media advertisement campaigns since they possibly received different rates. Although our calculations may not be exact, our figures were based on official rates provided by media outlets, meriting a solid foundation for PMI’s likely expenditures. In any case, considering we only calculated expenditure for one type of advertisement, it is unlikely we overestimated PMI’s expenditures and more likely underestimated them. On top of calculable advertisement expenses, we were unable to quantify some, such as for digital and online marketing, and other extensive expenses PMI likely incurred for more classical lobbying^17^.

## CONCLUSIONS

We provide indications PMI dominates the TA printed press market in Switzerland. Moreover, data show their print advertisement waves coincided with key moments of parliamentary discussions about now-adopted tobacco product legislation. Since PMI-related print media advertisements appeared during key moments of parliamentary discussions about future tobacco product laws, PMI possibly attempted to influence the public and politicians. Our advertisement monitoring methodology application helps us understand and explain contextual conditions of health in Switzerland. We recommend that future research and monitoring continue and suggest that our advertisement monitoring methodology will be useful for strengthening tobacco control policymaking. Finally, raising awareness of tobacco industry tactics and implementing a comprehensive ban on tobacco advertising in Switzerland are crucial public health efforts. Recognizing the media’s role in shaping public and policy agendas underscores the importance of strategic media engagement in tobacco control campaigns.

## Data Availability

The data supporting this research are available from the authors on reasonable request.
